# Immunogenetic markers associated with a naturally acquired humoral immune response against an N-terminal antigen of *Plasmodium vivax* merozoite surface protein 1 (PvMSP-1)

**DOI:** 10.1186/s12936-016-1350-2

**Published:** 2016-06-03

**Authors:** Gustavo Capatti Cassiano, Adriana A. C. Furini, Marcela P. Capobianco, Luciane M. Storti-Melo, Maria E. Almeida, Danielle R. L. Barbosa, Marinete M. Póvoa, Paulo A. Nogueira, Ricardo L. D. Machado

**Affiliations:** Department of Biology, São Paulo State University, São José Do Rio Preto, São Paulo Brazil; Department of Skin, Infectious and Parasitic Diseases, São José do Rio Preto Medical School, São José Do Rio Preto, São Paulo Brazil; Department of Biology, Federal University of Sergipe, São Cristóvão, Sergipe Brazil; Leônidas and Maria Deane Institute, Oswaldo Cruz Foundation, Manaus, Amazonas Brazil; Laboratory of Malaria Basic Research, Division of Parasitology, Evandro Chagas Institute, Belém, Pará Brazil

**Keywords:** *Plasmodium vivax*, MSP-1, ICB2-5, Immunogenetics, Antibodies

## Abstract

**Background:**

Humoral immune responses against proteins of asexual blood-stage malaria parasites have been associated with clinical immunity. However, variations in the antibody-driven responses may be associated with a genetic component of the human host. The objective of the present study was to evaluate the influence of co-stimulatory molecule gene polymorphisms of the immune system on the magnitude of the humoral immune response against a *Plasmodium vivax* vaccine candidate antigen.

**Methods:**

Polymorphisms in the *CD28*, *CTLA4*, *ICOS*, *CD40*, *CD86* and *BLYS* genes of 178 subjects infected with *P. vivax* in an endemic area of the Brazilian Amazon were genotyped by polymerase chain reaction-restriction fragment length polymorphism (PCR-RFLP). The levels of IgM, total IgG and IgG subclasses specific for ICB2-5, i.e., the N-terminal portion of *P. vivax* merozoite surface protein 1 (PvMSP-1), were determined by enzyme-linked immuno assay. The associations between the polymorphisms and the antibody response were assessed by means of logistic regression models.

**Results:**

After correcting for multiple testing, the IgG1 levels were significantly higher in individuals recessive for the single nucleotide polymorphism rs3116496 in *CD28* (*p* = 0.00004). Furthermore, the interaction between *CD28* rs35593994 and *BLYS* rs9514828 had an influence on the IgM levels (*p* = 0.0009).

**Conclusions:**

The results of the present study support the hypothesis that polymorphisms in the genes of co-stimulatory components of the immune system can contribute to a natural antibody-driven response against *P. vivax* antigens.

**Electronic supplementary material:**

The online version of this article (doi:10.1186/s12936-016-1350-2) contains supplementary material, which is available to authorized users.

## Background

According to the World Health Organization, there were an estimated 200 million cases of malaria in 2014 and an estimated 584,000 resulting deaths worldwide [1]. Among the five species that can cause malaria in humans, *Plasmodium vivax* is the most widely distributed, accounting for most of the cases of malaria in South and Southeast Asia, Latin America and Oceania; there are an estimated 2.5 billion people living in areas at risk of transmission of the disease [1, 2]. Furthermore, although vivax malaria has not been considered life-threatening for a long time, reports of severe cases associated with *P. vivax* have been increasingly numerous [3].

The blood stage of the *Plasmodium* lifecycle is responsible for the pathology associated with malaria. In this stage, merozoites released from schizont-infected erythrocytes invade non-infected erythrocytes, resulting in their destruction and the release of more merozoites into the bloodstream. During this brief extracellular period, these free merozoites are exposed to the host immune system, and proteins that are critical for parasite invasion are, therefore, important targets for the development of malaria vaccines. Merozoite surface proteins (MSPs) are among the most studied, especially MSP-1, which is necessary for merozoite attachment to erythrocytes [4] and normal parasite development [5].

The most widely accepted structure of the *P. vivax* merozoite surface protein 1 (PvMSP-1) gene indicates that it consists of six polymorphic blocks (blocks 2, 4, 6, 8, 10 and 12) flanked by seven conserved blocks (blocks 1, 3, 5, 7, 9, 11 and 13) [6]. By analysing the primary structure of PvMSP-1, several seroepidemiological studies have been performed to evaluate its immunogenic potential [7–11]. Although the C-terminal portion of the protein (PvMSP-1_19_) is the most immunogenic [7, 9, 12], a number of studies showed high prevalence of IgG against N-terminal PvMSP-1 in individuals exposed to *P. vivax* [9, 10, 13, 14]. Furthermore, specific IgG3 antibodies against the N-terminal portion of PvMSP-1 have been associated with clinical protection in two riverine communities of the Brazilian Amazon [9, 11], similar to that observed in *P. falciparum* infection, where persistence of antibodies IgG3 against N-terminal of MSP-1 was related with prolongation time without malaria [15]. In fact, antibodies specific for the asexual blood stage are thought to be involved in clinical protection against malaria vivax. Longitudinal cohort studies have shown correlations between magnitude of antibody responses to *P. vivax* merozoite antigens and protection from malaria [11, 16–18]. Due to the inability in maintaining *P. vivax* in continuous in vitro culture, it is difficult to define the role of antibodies to this species, but few evidences support that it may be related to inhibition of merozoite invasion [19, 20]. Furthermore, complement and FcR mediated mechanisms seem to be important in antibody-mediated protection [21].

The development of an adequate immune response depends on the fine regulation of lymphocyte activation. For this, in general, lymphocytes require two activation signals. The first signal is antigen-specific, whereas the second signal, called co-stimulation, is generated by the interaction between the surface molecules of T cells and those of antigen-presenting cells, including B cells. The interaction between CD28 and its ligands, CD80 and CD86, provides the strongest costimulatory signal for T-cell proliferation, whereas CTLA-4 is a negative regulator that plays a key role in T cell homeostasis and in central tolerance [22]. Another member of the CD28 family, Inducible co-stimulator (ICOS), is an important immune regulatory molecule that participates in T-cell activation and T-cell dependent B-cell responses [23, 24]. CD40 is presented on the surface of B-cells and the CD40-CD40L interaction is the major costimulatory signal for B cells to mount a humoral response [25]. B lymphocyte stimulator (BLYS) is produced mainly by innate immune cells and is needed to provide signals for B cell survival and proliferation [26]. Considering the importance of these molecules in development of immune response and because there are currently multiple lines of evidence showing that the genes involved in the immune response can influence antibody production during malaria infection [27–34], the authors hypothesised that polymorphisms in the genes of the co-stimulatory molecules CD28, CTLA-4, ICOS, CD86, CD40 and BLYS are involved in the magnitude of the naturally acquired antibody-driven response against N-terminal PvMSP-1 in individuals infected with *P. vivax* in the Brazilian Amazon.

## Methods

### Study area and subjects

The present study was conducted in the municipality of Goianésia do Pará, Pará state, Brazil, which is a constituent of the Brazilian Amazon region, where *P. vivax* is responsible for more than 80 % of all malaria cases [35]. Details of the study area have been described elsewhere [36]. Cross-sectional surveys conducted from February 2011 to August 2012 were used to recruit 178 subjects (125 men and 53 women) presenting with classic symptoms of malaria, who sought treatment at the medical service of the municipality; the subjects had an average age of 29.8 years (varying from 14 to 68 years). Exclusion criteria included children under 10 years old, pregnancy, related individuals and anti-malarial treatment within the previous seven days. Diagnosis was performed by microscopy (thick film), and infection with *P. vivax* was subsequently confirmed by nested polymerase chain reaction (PCR) [37]. The geometric mean of parasiatemia was 718.4 parasites/mm^3^ (95 % CI 487.2–1059.2). All patients with microscopically confirmed infections were given standard treatment of 25 mg/kg of chloroquine in 3 days plus 0.5 mg/kg of primaquine during seven days. All participants were submitted to a questionnaire to obtain epidemiological information. The length of residence in the municipality varied between one month and 37 years (median of 5 years), and 91.9 % of the individuals had previously contracted malaria. Samples from 40 malaria-naive individuals living in a non-endemic area (São José do Rio Preto, Brazil), who had never been to areas of malaria transmission, were used as controls.

Peripheral venous blood (~10 mL) was collected in EDTA tubes, and plasma samples were obtained by centrifugation and stored at −20 °C. The protocol of the present study was approved by the Research Ethics Committee of the Medical School of São José do Rio Preto (CEP/FAMERP no. 4599/2011) and by the health authorities of the municipality of Goianésia do Pará. All participants or their legal guardians signed an informed consent form.

### Genotyping

DNA was extracted from peripheral blood samples with an Easy-DNA™ extraction kit (Invitrogen, California, USA). All single nucleotide polymorphisms (SNPs) were genotyped by PCR-restriction fragment length polymorphism (RFLP). The SNPs −*372G* > *A* and +*17T* > *C* in *CD28* (rs35593994 and rs3116496, respectively), −*318C* > *T* and +*49A* > *G* in *CTLA4* (rs5742909 and rs231775, respectively), +*1564T* > *C* in *ICOS* (rs4404254), +*1057G* > *A* in *CD86* (rs1129055), −*1C* > *T* in *CD40* (rs1883832), and −*871C* > *T* in *BLYS* (rs9514828) were genotyped according to previously described protocols [36, 38, 39]. Primer sequences, restriction enzymes, and the restriction fragments obtained of each SNP are presented in Additional file 1. PCR for the identification of −*1722T* > *C* and −*1577G* > *A* in *CTLA4* (rs733618 and rs11571316, respectively) was performed with a final sample volume of 25 µL containing 1X Buffer (20 mM Tris-HCl pH 8.4, 50 mM KCl), 1.5 mM MgCl_2_, 0.2 mM of each dNTP, 0.6 pmol of each primer, and 0.5 U Platinum Taq DNA Polymerase (Invitrogen, São Paulo, Brazil). The primers 5′ CTTCATGCCGTTTCCAACTT 3′ and 5′ CCTTTTCTGACCTGCCTGTT 3′ were used for the −*1722T* > *C* genotyping, whereas 5′ CTTCATGCCGTTTCCAACTT 3′ and 5′ ATCTCCTCCAGGAAGCCTCTT 3′ were used to identify −*1577G* > *A*. Amplifications were performed under the following conditions: first step of 5 min at 94 °C, 35 cycles for 30 s at 94 °C, 30 s at 52 °C and 1 min at 72 °C, and a final step for 10 min at 72 °C. The PCR products of −*1722T* > *C* and −*1577G* > *A* were digested with the enzymes *Bbv*I and *Mbo*II (Fermentas, Vilnius, Lithuania), respectively. Electrophoresis was performed in 2.5 % agarose and stained with GelRed™ (Biotium, Hayward, USA) with the exception of the rs5742909 and rs1883832 SNPs, which were performed in 12.5 % polyacrylamide gel after staining with ethidium bromide, and visualized in a UV transilluminator.

### Antigen and antibody determination

The glutathione-S-transferase (GST)-tagged recombinant ICB2-5 protein corresponds to the amino acids 170-675 of the N-terminal portion of MSP-1 from the Belém strain of *P. vivax*. The ICB2-5-GST fusion protein was purified on a glutathione-Sepharose 4B column (Amersham Pharmacia), and the protein content was assessed with the Bio-Rad Protein Assay Kit I (Bio-Rad Laboratories, Inc.) [7]. The levels of IgM and IgG subclasses specific for ICB2-5 were measured as described previously with some modifications [7]. The plates were coated with GST-tagged ICB2-5 and GST alone, and all tests were done in duplicate. Briefly, 50 µL of ICB2-5-GST or GST alone (as a control) was coated on ELISA plates at 4 µg/mL (Costar, Corning Inc., New York, USA) and incubated overnight at 4 °C. After washing and blocking the plates with 0.05 % bovine serum albumin-PBS, 50 µL of plasma diluted 1/100 was added to the wells in duplicate and incubated for 1 h at 37 °C. Then, 50 µL of peroxidase-conjugated anti-human IgG or IgM antibodies (KPL, Maryland, USA) diluted 1/1000 were added to each well for the detection of total IgG or IgM, respectively. IgG subclasses were detected using mouse monoclonal antibodies specific for each isotype (Sigma, Missouri, USA), diluted according to the tested subclass (1/3000 for IgG1 clone HP6001; and IgG3 clone HP6050, 1/2500 for IgG2 clone HP6014, and 1/5000 for IgG4 clone HP6025). The immobilized monoclonal antibodies were detected with peroxidase-conjugated anti-mouse antibodies for 1 h at 37 °C (Sigma, Missouri, USA). Subsequently, the plates were washed and developed in the dark with 50 µL TMB substrate diluted 1/50 with 0.1 M phosphate-citrate buffer, pH 5.0, containing 0.03 % H_2_O_2_. The reaction was interrupted after 10 min by adding 50 µL of 2 N H_2_SO_4_ to each well and read at 450 nm. The determination of positivity for anti ICB2-5 was calculated as described previously, with some modifications [9]. Brief, firstly the average OD was calculated for each individual, and serum was considered positive if GST-tagged ICB2-5 OD was equal to or greater than cut off for this same protein, measured with sera of individuals who never had a past history of malaria. To confirm the positivity of a serum for anti ICB2-5, the average of GST-tagged ICB2-5 OD was calculated to exclude reactivity against GST. For each serum, the GST-cut off was calculated as average of GST OD plus twice the standard deviations (SD). The positivity for anti ICB2-5 was determined when average of GST-tagged ICB2-5 OD was equal to or greater than its GST-cut off. The results are expressed as the reactivity index (RI), which was calculated by dividing the OD of the sample by the cut-off value. Only the samples that were positive for total IgG (RI > 1) were tested for IgG subclasses.

### Statistical analysis

The associations between the SNP genotypes and the antibody response were analysed by means of a logistic regression model, controlling for potential confounders, such as age and gender. Different genetic models (codominant, dominant, recessive and log-additive) were tested with the ‘SNPSassoc’ package [40] implemented for the R statistical software (version 3.1.1). A Bonferroni correction was used to adjust for multiple testing, and the significance was set at *p* < 0.005 (0.05/10). The sample size was calculated using GWAPower software [41]. The current sample size (n = 178) had 80 % power to detect a variant with about 6 % heritability. Since only 90 samples were assessed for IgG subclasses, the power to detect the same heritability was about 42 %. Genotypic deviations from the Hardy–Weinberg equilibrium (HWE) were evaluated by using the exact test described by Wigginton et al. [42]. Statistically significant differences between the means of continuous variables were assessed with one-way analysis of variance (ANOVA). Correlation coefficients of the antibody levels were estimated with the Pearson correlation. Interactions between SNP pairs were also assessed with the ‘SNPassoc’ package, which determines the effects of interaction by means of log-likelihood ratio tests (LRTs).

Haplotype blocks were determined with Haploview 4.2 using the solid spine of linkage disequilibrium (LD) method [43], and the degree of LD between SNPs was estimated with the D’ parameter. The R package ‘haplo.stats’ [44] was used for association tests between haplotypes and antibody levels by means of the ‘haplo.glm’ function, which performs a regression that estimates the effect of each haplotype compared to a reference haplotype by means of a general linear model.

## Results

### Serological data and single-marker associations

Of the 178 subjects analysed, all infected with *P. vivax*, 90 (50.6 %) exhibited IgG (RI > 1), whereas 53 (29.8 %) exhibited IgM specific for ICB2-5. The antibody levels were higher for IgG1 (mean = 1.08) compared to those of the other subclasses (means of 0.91, 0.79 and 0.79 for IgG2, IgG3 and IgG4, respectively) (*p* < 0.001, ANOVA). The highest correlations between levels of IgG subclasses were found for IgG1 and IgG2 (r = 0.48) and IgG1 and IgG3 (r = 0.43) (Additional file 2). The levels of ICB2-5-specific antibodies were not influenced by previous exposure to malaria (age, length of residence in the studied area, and previous episodes of malaria) (Additional file 3), except for IgG2, which exhibited higher levels in individuals who reported to have had less than five previous episodes of malaria, compared to those who reported to have had more than five previous episodes [mean (confidence interval): 0.97 (0.84–1.10) vs. 0.88 (0.76–0.99), *p* = 0.01, ANOVA].

 The allelic and genotype frequencies of all the analysed SNPs are listed in Table 1 and Additional file 4. The allele frequencies of these SNPs were previously presented in a larger set of samples (with exception of SNPs rs733618 and rs11571316) [45]. The success rate of SNP genotyping was 100 % for eight of the studied SNPs, whereas rs733618 and rs11571316 were successfully genotyped in 88.2 and 82 % of the samples, respectively, and no deviation from HWE was observed (all *p* value >0.1). A summary of the statistics for all the evaluated SNPs is listed in Table 1 and Additional file 4. There is not significant association between the polymorphisms and parasitaemia (all *p* value >0.07, Kruskal–Wallis test, data not show). Three SNPs were significantly associated with the humoral immune response against *P. vivax* ICB2-5. IgM levels were associated with rs35593994 in *CD28*; individuals with the *GG* genotype had lower antibody levels (mean 0.67 vs. 0.88, *p* = 0.03). Based on a recessive model, individuals with a *CC* genotype for rs3116496 in *CD28* exhibited higher IgG1 levels with respect to the other genotypes (mean 3.13 vs. 1.04, *p* = 0.00004). Individuals with a *TC* genotype for rs733618 in *CTLA4* exhibited higher IgG2 levels with respect to homozygous individuals (mean 1.18 vs. 0.87, *p* = 0.04). However, after correction for multiple testing, only association that remained significant was the one with *CD28* (re3116496).

**Table 1:** Levels of *Plasmodium* *vivax* ICB2-5-specific IgM, IgG and IgG subclasses with respect to the analysed genotypes from individuals infected with *P. vivax*

SNP	Gene	Genotypes	Genotypes (n^a^)	Genotypes (n^b^)	IgG (RI)	IgG1 (RI)	IgG2 (RI)	IgG3 (RI)	IgG4 (RI)	IgM (RI)
rs35593994	*CD28*	GG/GA/AA	86/80/12	42/39/8	1.02/1.03/1.11	1.11/1.10/0.89	0.92/0.93/0.80	0.76/0.86/0.64	0.77/0.83/0.74	0.66/0.86/1.13^c^
rs3116496	*CD28*	TT/TC/CC	118/54/6	60/27/2	1.04/1.02/0.91	1.06/0.98/3.13^d^	0.91/0.92/0.95	0.82/0.72/0.75	0.82/0.75/0.67	0.83/0.70/0.65
rs733618	*CTLA4*	TT/TC/CC	135/20/2	72/7/1	1.06/0.89/1.05	1.11/0.97/0.81	0.87/1.18/0.64^e^	0.80/0.66/0.57	0.80/0.68/0.62	0.82/0.72/1.25
rs11571316	*CTLA4*	GG/GA/AA	59/75/12	29/39/4	1.04/1.03/0.97	1.17/1.10/0.84	0.96/0.95/0.68	0.74/0.86/0.56	0.73/0.82/0.66	0.81/0.75/0.79
rs5742909	*CTLA4*	CC/CT/TT	151/27/0	75/14/0	1.15/1.01/–	1.04/1.29/–	0.91/0.93/–	0.80/0.76/–	0.80/0.78/–	0.79/0.78/–
rs231775	*CTLA4*	AA/AG/GG	75/84/19	40/41/8	1.07/1.00/1.01	0.96/1.22/0.98	0.87/0.98/0.82	0.78/0.82/0.70	0.79/0.79/0.81	0.79/0.75/0.89
rs4675378	*ICOS*	TT/TC/CC	77/73/27	35/38/16	1.00/1.05/1.09	1.08/1.12/0.99	0.86/0.94/0.96	0.73/0.90/0.67	0.80/0.78/0.80	0.80/0.73/0.85
rs1129055	*CD86*	GG/GA/AA	110/60/8	53/31/5	1.00/1.08/1.15	1.08/1.09/1.06	0.93/0.90/0.93	0.78/0.82/0.70	0.82/0.76/0.70	0.81/0.73/0.90
rs1883832	*CD40*	CC/CT/TT	135/37/6	65/20/4	1.01/1.06/1.25	1.11/1.03/0.86	0.92/0.95/0.68	0.81/0.77/0.65	0.81/0.75/0.79	0.80/0.69/1.13
rs9514828	*BLYS*	CC/CT/TT	103/62/13	48/35/6	0.99/1.07/1.15	0.99/1.23/0.98	0.91/0.94/0.83	0.82/0.69/1.11	0.76/0.81/0.92	0.73/0.83/0.95

^a^Number of individuals evaluated for ICB2-5-specific IgM and total IgG (*n* = 178)

^b^Number of individuals evaluated for ICB2-5-specific IgG subclasses (*n* = 90)

^c^Significant according to the dominant model (*p* = 0.033)

^d^Significant according to the recessive model (*p* = 0.00004)

^e^Significant according to the overdominant model (*p* = 0.042)

### Linkage disequilibrium analysis and haplotype association

**Fig. 1:**
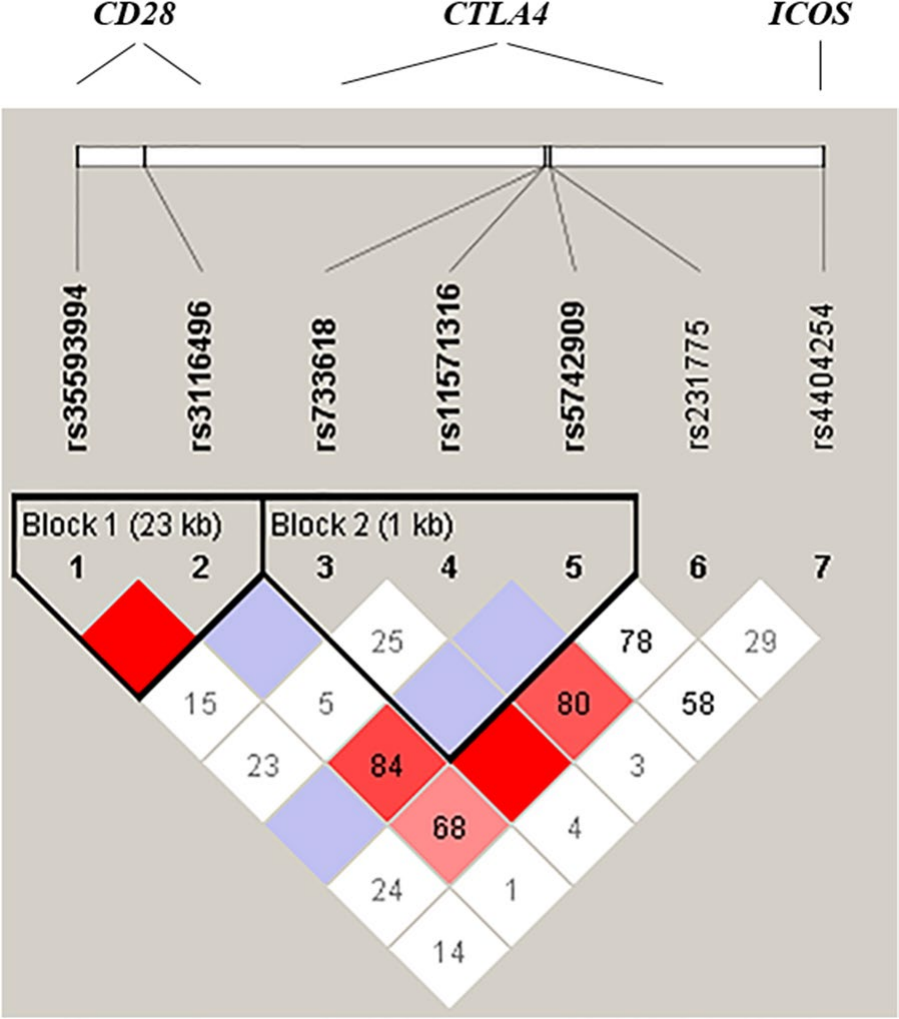
Linkage disequilibrium of investigated polymorphisms in region 2q33, which comprises genes *CD28*, *CTLA4* and *ICOS*. The *numbers* within the *squares* represent the D’ value, expressed as per cent. *Empty squares* represent a D’ value of 1, which indicates complete linkage disequilibrium. *Darker-shaded squares* represent pairs with an LOD score = 2, *light grey squares* represent D’ = 1 and LOD < 2, and *white squares* represent LOD < 2 and D’ < 1. *Two blocks* were identified by means of the *solid* spine of linkage disequilibrium method [37]. Linkage Disequilibrium map was generated by Haploview 4.2

Linkage disequilibrium analysis was performed for all possible pairwise combinations of seven SNPs that were analysed across 255 kb in the 2q33 chromosomal region, which encompasses *CD28*, *CTLA4* and *ICOS*. Two haplotype blocks were defined using the criterion described by Barret et al. [43] (Fig. 1). Three haplotypes of block 1, which contains rs35593994 and rs3116496 in *CD28*, were built, with frequencies varying between 18.5 and 52.2 %, and for block 2, which contains rs733618, rs11571316 and rs5742909 in the promoter region of *CTLA4*, four haplotypes were built, with frequencies varying between 6.3 and 51.8 %. The effects of each haplotype on the antibody levels were estimated and are shown in Table 2. The AT haplotype in block 1 was significantly associated with a 20 % increase in IgM levels (*p* = 0.027) with respect to the reference haplotype (specifically, the most frequent haplotype, i.e., CT). However, no association remained significant after correction for multiple testing.

**Table 2:** Haplotype frequencies and their association with levels of ICB2-5-specific antibodies

Block	Haplotype	Frequency	IgG		IgG1		IgG2		IgG3		IgG4		IgM	
			∆%	*p*	∆%	*p*	∆%	*p*	∆%	*p*	∆%	*p*	∆%	*p*
Block 1	G-T	0.522	Reference											
	A-T	0.292	2 (−9; 13)	0.72	−3 (−27; 21)	0.80	−3 (−16; 9)	0.61	0 (−15; 14)	0.95	0 (−10; 10)	0.99	20 (2; 38)	*0.027*
	G-C	0.185	−3 (−16; 9)	0.59	21 (−9; 51)	0.17	0 (−15; 16)	0.96	−8 (−27; 10)	0.38	−7 (−19; 5)	0.26	−6 (−27; 15)	0.58
Block 2	T-G-C	0.518	Reference											
	T-A-C	0.330	−2 (−14; 10)	0.72	−4 (−32; 25)	0.81	−9 (−23; 5)	0.21	2 (−16; 19)	0.85	3 (−9; 16)	0.59	−7 (−28; 13)	0.48
	T-G-T	0.076	12 (−7; 31)	0.22	49 (−23; 121)	0.18	3 (−21; 27)	0.83	−4 (−34; 25)	0.77	−3 (−23; 17)	0.77	−3 (−34; 27)	0.84
	C-G-C	0.063	−17 (−38; 11)	0.27	−10 (−70; 51)	0.75	−14 (−44; 17)	0.38	−14 (−52; 23)	0.45	−11 (−36; 14)	0.38	−23 (−54; 9)	0.16

The effects of each haplotype are relative to the most frequent haplotype, which was used as a reference. ∆% indicates changes relative to the antibody levels compared to reference haplotypes, with 95 % confidence intervals. Block 1 refers to the SNPs rs35593994 and rs3116496, and block 2 refers to rs733618, rs11571316 and rs5742909

### Interaction analysis

**Fig. 2:**
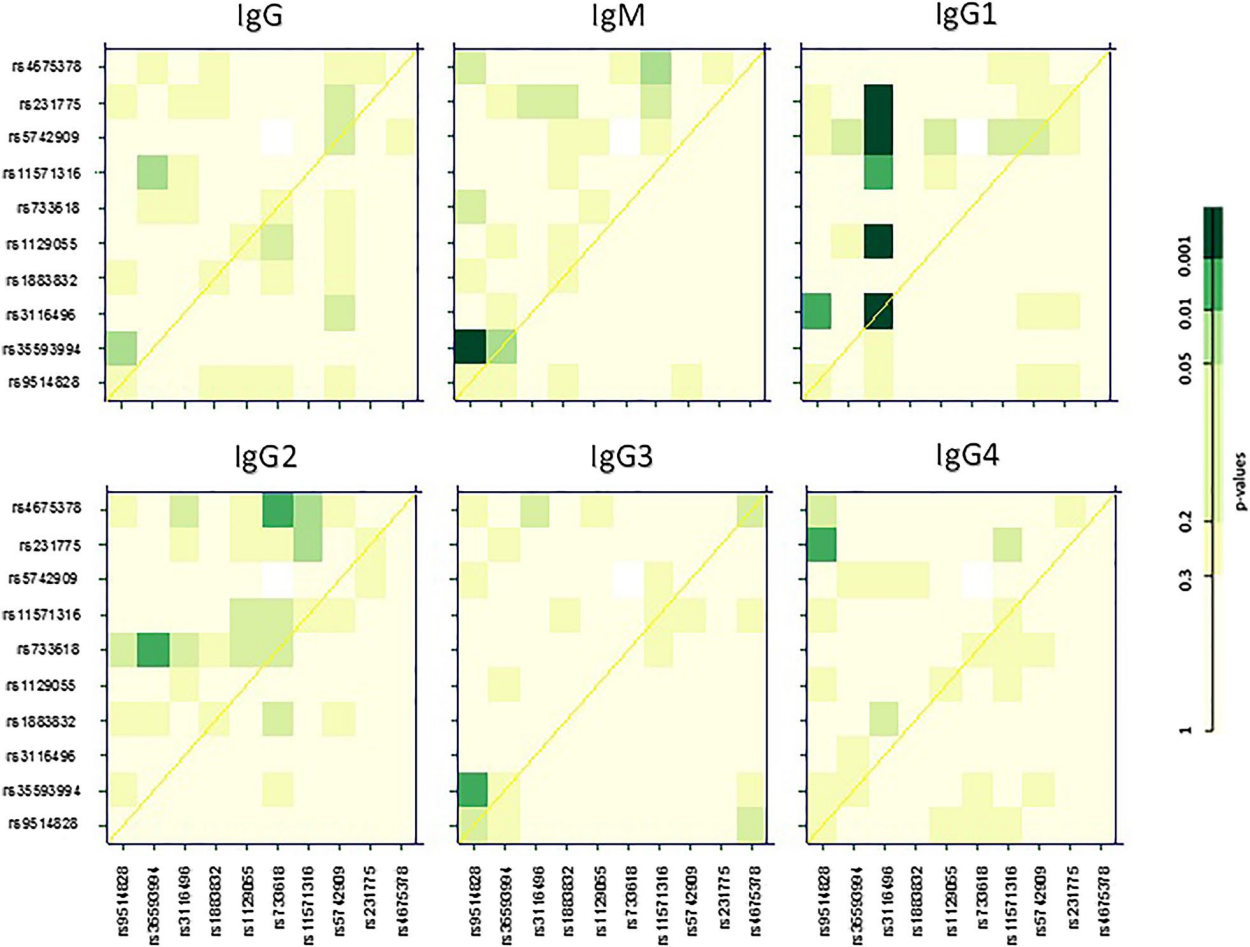
Interaction between SNPs in the codominant model. The diagonal contains the *p* values of the likelihood ratio test (LRT) for the effect of each SNP individually. The *upper triangle* in the matrix contains the *p* values for the interaction log-LRT. The *lower triangle* contains the *p* values from LRT comparing the two-SNP additive likelihood to the best finding of the single-SNP model [35]

Using the R project package ‘SNPassoc’ [40], the study explored all pairwise SNP−SNP interactions under the codominant model. These interactions are represented graphically in Fig. 2, in which the upper part contains the *p* values for the interaction LRT. Although several significant associations involving rs3116496 in *CD28* and other SNPs (rs1129055, rs5742909 and rs231775) with IgG1 levels were observed, the level of significance was below that found for rs3116496 individually (all *p* values >0.0001). However, the interaction involving rs35593994 in *CD28* with rs9514828 in *BLYS* was associated with the IgM levels (*p* = 0.0009). A regression analysis was performed to confirm this interaction, and the results of significant associations involving the above two SNPs are listed in Table 3.

**Table 3:** Significant interactions between the SNPs rs35593994 and rs9514828 and the levels of IgM specific for ICB2-5

*CD28* (rs35593994)	*BLYS* (rs9514828)	IgM (RI^a^)	∆^b^	95 % CI	*p* ^c^
GG	CC	0.503	Reference		
GG	CT	1.025	0.523	(0.195–0.850)	0.002
GA	CC	0.916	0.413	(0.126–0.702)	0.005
GA	TT	1.441	0.939	(0.395–1.483)	0.0009

^a^Antibody levels are expressed as the mean of the reactivity index (RI)

^b^Difference of the RI mean

^c^Based on a logistic regression model using individuals exhibiting wild type genotypes (*GG* and *CC*) as a reference

## Discussion

The main strategies used for malaria control are based on prompt diagnosis and treatment and on vector control. However, new resistant parasite strains arise as new drugs are applied, and vector control is also encountering great challenges due to the growing resistance to insecticides, thus justifying research on the development of a vaccine that is effective against malaria. Characterisation of the naturally acquired immune response in different populations is a useful tool for the identification of molecules that can be targeted by anti-malarial vaccines.

The present study was to evaluate the naturally acquired immune response against the N-terminal portion of PvMSP-1. Although the C-terminal portion (MSP-1_19_) is considered to be the most immunogenic region of the protein [7, 9, 12], there is evidence suggesting that antibodies targeting the N-terminal portion of MSP-1 provide clinical protection during infections with both *P. falciparum* [46, 47] and *P. vivax* [9, 11]. The results of the IgG-mediated humoral immune response showed that ICB2-5 was detected by just over half of the studied individuals (50.6 %). This prevalence of responders is similar to that found in individuals infected with *P. vivax* in other places of the Brazilian Amazon [8, 11]. A small percentage (~10 %) of the individuals who reported having had several previous episodes of malaria exhibited high IgM levels, but no IgG was evidenced, suggesting that class switching from IgM to IgG may be impaired. This observation has been reported in previous studies [8, 48], and Soares et al. [8] suggested that this impaired switch from IgM to IgG may be related to deficient CD40/CD40-L interactions. Thus, the authors evaluated whether co-stimulatory molecule gene polymorphisms are involved in the delayed class switch from IgM to IgG. However, no association was observed.

Regarding IgG subclasses, higher levels of IgG1 were found compared to the other IgG subclasses, which is in contrast to previous studies showing a predominance of IgG3 specific for ICB2-5 [8, 9, 11]. Although there is still no consensus regarding the role of antibody subclasses in clinical protection, it has been suggested that only the cytophilic subclasses, i.e., IgG1 and IgG3, are protective [49, 50]. Two longitudinal studies performed in the Brazilian Amazon, specifically, one in Portuchuello, near Porto Velho, and the other in a community of Rio Pardo, Amazonas state, have observed that ICB2-5-specific antibodies were associated with clinical protection against malaria caused by *P. vivax* and that IgG3 was detected in all asymptomatic individuals, whereas most symptomatic patients exhibited no IgG3 [9, 11]. If the above results were to be extrapolated to Goianésia do Pará, the site of this study, the fact that a passive collection was performed on patients exhibiting symptoms could explain the low IgG3 levels found. Furthermore, some studies have also shown that IgG2 may be associated with protection. Deloron *et* al. [51] found an association between high IgG2 levels and low risk of acquiring an infection by *P. falciparum*. Although the design of the present study does not allow for the association of the prevalence and levels of clinically protective antibodies, higher IgG2 levels were observed in subjects who reported having had fewer cases of malaria.

The development of an immune response against *Plasmodium* species is a complex process, and one of the main issues is understanding why individuals differ in their immune responses against the parasite. Thus, the objective of the present study was to investigate the influence of co-stimulatory molecule gene polymorphisms on the production of antibodies specific for an important *P. vivax* vaccine candidate antigen. The most important result was that *CD28* rs3116496 was associated with levels of IgG1 specific for ICB2-5. In addition to the important role of CD28 in T cell activation, the binding of this receptor to its ligands CD80 and CD86 on the surface of B cells provides bidirectional signals that appear to be important for IgG production by B cells [52]. Thus, CD28 may be involved in the immune response against malaria. In fact, *CD28* knockout mice infected with *Plasmodium chabaudi* were unable to resolve the infection, maintaining low levels of parasitaemia for weeks after infection [53, 54]. Furthermore, treatment of wild type mice with monoclonal anti-CD86 antibodies abolished IL-4 production and was significantly associated with reduced levels of *P. chabaudi*-specific IgG1 [55].

In the present study, individuals exhibiting the *T* allele for rs3116496 in *CD28* were found to be associated with reduced IgG1 levels. Although the biological functions of this SNP, which is located in the third intron of the gene, are still unknown, it is located near a splice site, at which point mutations can induce abnormal splicing, thus affecting protein expression [56]. The relationship between rs3116496 in *CD28* and susceptibility to several diseases has already been evaluated, and significant associations have been found in type 1 diabetes [57], cervical [58] and breast cancer [59], and rheumatoid arthritis [60]. However, the role of this polymorphism in malaria has not yet been assessed, and even if the presence of the *T* allele, which is associated with lower levels of ICB2-5-specific IgG1, were implicated in higher susceptibility to vivax malaria, further elucidation would still be necessary.

Although it seems likely that immunity against malaria is affected by several genes, the influence of combined polymorphisms is rarely ever investigated. The present study performed analyses of interactions between the SNP pairs and found that *CD28* rs35593994 and *BLYS* rs9514828 together were associated with levels of IgM specific for ICB2-5, especially in individuals with *GA* and *TT* genotypes, respectively. Specifically, they were associated with a ~180 % increase in IgM levels compared to that of wild type genotype individuals. Although the biological mechanisms underlying this interaction are still unknown, CD28 and BLyS may be involved in the production of memory B cells and antibody isotype class switching [61, 62]. Both polymorphisms, i.e., *CD28* rs35593994 and *BLYS* rs9514828, are located in the 5′ regulatory region of the gene; thus, differential expression of these genes may change the regulation of the B cells involved in the production of ICB2-5-specific antibodies. Liu et al. [63] have shown that despite the generation of memory B cells as a response to vaccination with MSP-1_19_, the function of these cells was nullified due to a lack of BLyS expression in dendritic cells from mice infected with *P. yoelii*.

Although it was observed the influence of these two SNPs in the antibody response to ICB2-5, most of the evaluated polymorphisms did not show any significant differences. In fact, variations in the immune response to malaria can be attributed to several factors, including the environment, previous exposure to malaria and immunogenicity of antigen. Thus, as a complex trait, it is likely that many polymorphisms have small effect in malaria immune response, but large sample size is required to detect it.

## Conclusions

In summary, several genes involved in the control of the immune response were investigated, entailing the production of antibodies against a *P. vivax* vaccine candidate antigen. Despite the growing number of studies evaluating human genetic factors that control the immune response to malaria [27–34, 64], it is likely that many genes responsible for the wide inter-individual variation observed in the immune response against malaria remain unknown; studies similar to the present work may help identify subjects who are more prone to respond to a specific vaccine [29]. Although the statistical evidence supporting the described associations is limited by the relatively small sample size and, although it is impossible to tell whether the SNPs studied here are truly causal or are in LD with the causal variants, the results suggest that genetic variations in *CD28* and interactions between polymorphisms in *BLYS* and *CD28* may be involved in the control of the immune response against vivax malaria.

## Additional files

10.1186/s12936-016-1350-2 Reaction conditions for the amplification and enzyme digestion of polymorphisms in the studied polymorphisms.
10.1186/s12936-016-1350-2 Correlation between ICB2-5-specific IgG, IgM and IgG subclasses in individuals infected with *Plasmodium vivax* living in an endemic area of the Brazilian Amazon.
10.1186/s12936-016-1350-2 IgM and IgG subclasses levels (RI) for ICB2-5 according to age, length of residence in the studied area and previous episode of malaria.
10.1186/s12936-016-1350-2 Association tests between genetic polymorphisms and *Plasmodium vivax* ICB2-5-specific antibody levels.

## References

[1] WHO (2013). World malaria report.

[2] Gething PW, Elyazar IR, Moyes CL, Smith DL, Battle KE, Guerra CA (2012). A long neglected world malaria map: *Plasmodium vivax* endemicity in 2010. PLoS Negl Trop Dis.

[3] Lacerda MV, Mourão MP, Alexandre MA, Siqueira AM, Magalhães BM, Martinez-Espinosa FE (2012). Understanding the clinical spectrum of complicated *Plasmodium vivax* malaria: a systematic review on the contributions of the Brazilian literature. Malar J.

[4] Goel VK, Li X, Chen H, Liu SC, Chishti AH, Oh SS (2003). Band 3 is a host receptor binding merozoite surface protein 1 during the *Plasmodium falciparum* invasion of erythrocytes. Proc Natl Acad Sci U S A.

[5] Child MA, Epp C, Bujard H, Blackman MJ (2010). Regulated maturation of malaria merozoite surface protein-1 is essential for parasite growth. Mol Microbiol.

[6] Putaporntip C, Jongwutiwes S, Sakihama N, Ferreira MU, Kho WG, Kaneko A (2002). Mosaic organization and heterogeneity in frequency of allelic recombination of the *Plasmodium vivax* merozoite surface protein-1 locus. Proc Natl Acad Sci U S A.

[7] Soares IS, Levitus G, Souza JM, Del Portillo HA, Rodrigues MM (1997). Acquired immune responses to the N- and C-terminal regions of *Plasmodium vivax* merozoite surface protein 1 in individuals exposed to malaria. Infect Immun.

[8] Soares IS, da Cunha MG, Silva MN, Souza JM, Del Portillo HA, Rodrigues MM (1999). Longevity of naturally acquired antibody responses to the N- and C-terminal regions of *Plasmodium vivax* merozoite surface protein 1. Am J Trop Med Hyg.

[9] Nogueira PA, Alves FP, Fernandez-Becerra C, Pein O, Santos NR, Pereira da Silva LH (2006). A reduced risk of infection with *Plasmodium vivax* and clinical protection against malaria are associated with antibodies against the N terminus but not the C terminus of merozoite surface protein 1. Infect Immun.

[10] Storti-Melo LM, Souza-Neiras WC, Cassiano GC, Taveira LC, Cordeiro AJ, Couto VS (2011). Evaluation of the naturally acquired antibody immune response to the Pv200 L N-terminal fragment of *Plasmodium vivax* merozoite surface protein-1 in four areas of the Amazon Region of Brazil. Am J Trop Med Hyg.

[11] Versiani FG, Almeida ME, Melo GC, Versiani FO, Orlandi PP, Mariúba LA (2013). High levels of IgG3 anti ICB2-5 in *Plasmodium vivax*-infected individuals who did not develop symptoms. Malar J.

[12] Riccio EK, Totino PR, Pratt-Riccio LR, Ennes-Vidal V, Soares IS, Rodrigues MM (2013). Cellular and humoral immune responses against the *Plasmodium vivax* MSP-1_19_ malaria vaccine candidate in individuals living in an endemic area in north-eastern Amazon region of Brazil. Malar J.

[13] Levitus G, Mertens F, Speranca MA, Camargo LM, Ferreira MU, del Portillo HA (1994). Characterization of naturally acquired human IgG responses against the N-terminal region of the merozoite surface protein 1 of *Plasmodium vivax*. Am J Trop Med Hyg.

[14] Fernandez-Becerra C, Sanz S, Brucet M, Stanisic DI, Alves FP, Camargo EP (2010). Naturally-acquired humoral immune responses against the N- and C-termini of the *Plasmodium vivax* MSP1 protein in endemic regions of Brazil and Papua New Guinea using a multiplex assay. Malar J.

[15] Cavanagh DR, Dodoo D, Hviid L, Kurtzhals JA, Theander TG, Akanmori BD (2004). Antibodies to the N-terminal block 2 of *Plasmodium falciparum* merozoite surface protein 1 are associated with protection against clinical malaria. Infect Immun.

[16] King CL, Michon P, Shakri AR, Marcotty A, Stanisic D, Zimmerman PA (2008). Naturally acquired Duffy-binding protein-specific binding inhibitory antibodies confer protection from blood-stage *Plasmodium vivax* infection. Proc Natl Acad Sci U S A.

[17] Cole-Tobian JL, Michon P, Biasor M, Richards JS, Beeson JG, Mueller I (2009). Strain-specific Duffy binding protein antibodies correlate with protection against infection with homologous compared to heterologous *Plasmodium vivax* strains in Papua New Guinean children. Infect Immun.

[18] Stanisic DI, Javati S, Kiniboro B, Lin E, Jiang J, Singh B (2013). Naturally acquired immune responses to *P. vivax* merozoite surface protein 3α and merozoite surface protein 9 are associated with reduced risk of *P. vivax* malaria in young Papua New Guinean children. PLoS Negl Trop Dis.

[19] Grimberg BT, Udomsangpetch R, Xainli J, McHenry A, Panichakul T, Sattabongkot J (2007). *Plasmodium vivax* invasion of human erythrocytes inhibited by antibodies directed against the Duffy binding protein. PLoS Med.

[20] Vicentin EC, Françoso KS, Rocha MV, Iourtov D, Dos Santos FL, Kubrusly FS (2014). Invasion-inhibitory antibodies elicited by immunization with *Plasmodium vivax* apical membrane antigen-1 expressed in *Pichia pastoris* yeast. Infect Immun.

[21] Beeson JG, Drew DR, Boyle MJ, Feng G, Fowkes FJ, Richards JS (2016). Merozoite surface proteins in red blood cell invasion, immunity and vaccines against malaria. FEMS Microbiol Rev.

[22] Gardner D, Jeffery LE, Sansom DM (2014). Understanding the CD28/CTLA-4 (CD152) pathway and its implications for costimulatory blockade. Am J Transplant.

[23] Coyle AJ, Gutierrez-Ramos JC (2004). The role of ICOS and other costimulatory molecules in allergy and asthma. Springer Semin Immunopathol.

[24] Simpson TR, Quezada SA, Allison JP (2010). Regulation of CD4 T cell activation and effector function by inducible costimulator (ICOS). Curr Opin Immunol.

[25] Chatzigeorgiou A, Lyberi M, Chatzilymperis G, Nezos A, Kamper E (2009). CD40/CD40L signaling and its implication in health and disease. BioFactors.

[26] Stadanlick JE, Cancro MP (2008). BAFF and the plasticity of peripheral B cell tolerance. Curr Opin Immunol.

[27] Carpenter D, Abushama H, Bereczky S, Färnert A, Rooth I, Troye-Blomberg M (2007). Immunogenetic control of antibody responsiveness in a malaria endemic area. Hum Immunol.

[28] Duah NO, Weiss HA, Jepson A, Tetteh KK, Whittle HC, Conway DJ (2009). Heritability of antibody isotype and subclass responses to *Plasmodium falciparum* antigens. PLoS ONE.

[29] Pandey JP, Morais CG, Fontes CJ, Braga EM (2010). Immunoglobulin GM 3 23 5,13,14 phenotype is strongly associated with IgG1 antibody responses to *Plasmodium vivax* vaccine candidate antigens PvMSP1-19 and PvAMA-1. Malar J.

[30] Afridi S, Atkinson A, Garnier S, Fumoux F, Rihet P (2012). Malaria resistance genes are associated with the levels of IgG subclasses directed against *Plasmodium falciparum* blood-stage antigens in Burkina Faso. Malar J.

[31] Dewasurendra RL, Suriyaphol P, Fernando SD, Carter R, Rockett K, Corran P (2012). Genetic polymorphisms associated with anti-malarial antibody levels in a low and unstable malaria transmission area in southern Sri Lanka. Malar J.

[32] Lima-Junior JC, Rodrigues-da-Silva RN, Banic DM, Jiang J, Singh B, Fabrício-Silva GM (2012). Influence of HLA-DRB1 and HLA-DQB1 alleles on IgG antibody response to the *P. vivax* MSP-1, MSP-3α and MSP-9 in individuals from Brazilian endemic area. PLoS ONE.

[33] Storti-Melo LM, da Costa DR, Souza-Neiras WC, Cassiano GC, Couto VS, Póvoa MM (2012). Influence of HLA-DRB-1 alleles on the production of antibody against CSP, MSP-1, AMA-1, and DBP in Brazilian individuals naturally infected with *Plasmodium vivax*. Acta Trop.

[34] Sabbagh A, Courtin D, Milet J, Massaro JD, Castelli EC, Migot-Nabias F (2013). Association of HLA-G 3’ untranslated region polymorphisms with antibody response against *Plasmodium falciparum* antigens: preliminary results. Tissue Antigens.

[35] Ministério da Saúde. Secretaria de Vigilância em Saúde [Ministry of Health. Department of Health Surveillance]. Malária. Resumo epidemiológico de malária no Brasil. 2013 [Malaria. Epidemiological report of malaria in Brazil. 2013]. Sistema de Informações de Vigilância Epidemiológica (SIVEP). 2013. http://portalweb04.saude.gov.br/sivep_malaria. Accessed 27 Dec 2013.

[36] Cassiano GC, Furini AC, Capobianco MP, Storti-Melo LM, Cunha MG, Kano FS (2016). Polymorphisms in B cell co-stimulatory genes are associated with IgG antibody responses against blood–stage proteins of *Plasmodium vivax*. PLoS ONE.

[37] Snounou G, Viriyakosol S, Jarra W, Thaithong S, Brown KN (1993). Identification of the four human malaria parasite species in field samples by the polymerase chain reaction and detection of a high prevalence of mixed infections. Mol Biochem Parasitol.

[38] Guzman VB, Morgun A, Shulzhenko N, Mine KL, Gonçalves-Primo A, Musatti CC (2005). Characterization of CD28, CTLA4, and ICOS polymorphisms in three Brazilian ethnic groups. Hum Immunol.

[39] Malheiros D, Petzl-Erler ML (2009). Individual and epistatic effects of genetic polymorphisms of B-cell co-stimulatory molecules on susceptibility to pemphigus foliaceus. Genes Immun.

[40] Gonzalez JR, Armengol L, Sole X, Guino E, Mercader JM, Estivill X (2007). SNPassoc: an R package to perform whole genome association studies. Bioinformatics.

[41] Feng S, Wang S, Chen CC, Lan L (2011). GWAPower: a statistical power calculation software for genome-wide association studies with quantitative traits. BMC Genet.

[42] Wigginton JE, Cutler DJ, Abecasis GR (2005). A note on exact tests of Hardy–Weinberg equilibrium. Am J Hum Genet.

[43] Barrett JC, Fry B, Maller J, Daly MJ (2005). Haploview: analysis and visualization of LD and haplotype maps. Bioinformatics.

[44] Sinnwell JP, Schaid DJ. Haplo.stats: Statistical analysis of haplotypes with traits and covariates when linkage phase is ambiguous. R package version 1.4.4. http://CRAN.R-project.org/package=haplo.stats (2014). Accessed 12 Dec 2014.

[45] Cassiano GC, Santos EJ, Maia MH, Furini Ada C, Storti-Melo LM, Tomaz FM (2015). Impact of population admixture on the distribution of immune response co-stimulatory genes polymorphisms in a Brazilian population. Hum Immunol.

[46] Conway DJ, Cavanagh DR, Tanabe K, Roper C, Mikes ZS, Sakihama N (2000). A principal target of human immunity to malaria identified by molecular population genetic and immunological analyses. Nat Med.

[47] Polley SD, Tetteh KK, Cavanagh DR, Pearce RJ, Lloyd JM, Bojang KA (2003). Repeat sequences in block 2 of *Plasmodium falciparum* merozoite surface protein 1 are targets of antibodies associated with protection from malaria. Infect Immun.

[48] Mertens F, Levitus G, Camargo LM, Ferreira MU, Dutra AP, Del Portillo HA (1993). Longitudinal study of naturally acquired humoral immune responses against the merozoite surface protein 1 of *Plasmodium vivax* in patients from Rondonia, Brazil. Am J Trop Med Hyg.

[49] Bouharoun-Tayoun H, Oeuvray C, Lunel F, Druilhe P (1995). Mechanisms underlying the monocyte-mediated antibody-dependent killing of *Plasmodium falciparum* asexual blood stages. J Exp Med.

[50] Nasr A, Hamid O, Al-Ghamdi A, Allam G (2013). Anti-malarial IgG subclasses pattern and FcγRIIa (CD32) polymorphism among pregnancy-associated malaria in semi-immune Saudi women. Malar J.

[51] Deloron P, Dubois B, Le Hesran JY, Riche D, Fievet N, Cornet M (1997). Isotypic analysis of maternally transmitted *Plasmodium falciparum*-specific antibodies in Cameroon, and relationship with risk of *P. falciparum* infection. Clin Exp Immunol.

[52] Rau FC, Dieter J, Luo Z, Priest SO, Baumgarth N (2009). B7-1/2 (CD80/CD86) direct signaling to B cells enhances IgG secretion. J Immunol.

[53] Rummel T, Batchelder J, Flaherty P, LaFleur G, Nanavati P, Burns JM (2004). CD28 costimulation is required for the expression of T-cell-dependent cell-mediated immunity against blood-stage *Plasmodium chabaudi* malaria parasites. Infect Immun.

[54] Elias RM, Sardinha LR, Bastos KR, Zago CA, da Silva AP, Alvarez JM (2005). Role of CD28 in polyclonal and specific T and B cell responses required for protection against blood stage malaria. J Immunol.

[55] Taylor-Robinson AW, Smith EC (1999). Modulation of experimental blood stage malaria through blockade of the B7/CD28 T-cell costimulatory pathway. Immunology.

[56] Baralle D, Baralle M (2005). Splicing in action: assessing disease causing sequence changes. J Med Genet.

[57] Ihara K, Ahmed S, Nakao F, Kinukawa N, Kuromaru R, Matsuura N (2001). Association studies of CTLA-4, CD28, and ICOS gene polymorphisms with type 1 diabetes in the Japanese population. Immunogenetics.

[58] Ivansson EL, Juko-Pecirep I, Gyllensten UB (2010). Interaction of immunological genes on chromosome 2q33 and IFNG in susceptibility to cervical cancer. Gynecol Oncol.

[59] Chen S, Zhang Q, Shen L, Liu Y, Xu F, Li D (2012). Investigation of CD28 gene polymorphisms in patients with sporadic breast cancer in a Chinese Han population in Northeast China. PLoS ONE.

[60] Ledezma-Lozano IY, Padilla-Martínez JJ, Leyva-Torres SD, Parra-Rojas I, Ramírez-Dueñas MG, Pereira-Suárez AL (2011). Association of CD28 IVS3 +17T/C polymorphism with soluble CD28 in rheumatoid arthritis. Dis Markers.

[61] Ferguson SE, Han S, Kelsoe G, Thompson CB (1996). CD28 is required for germinal center formation. J Immunol.

[62] Avery DT, Kalled SL, Ellyard JI, Ambrose C, Bixler SA, Thien M (2003). BAFF selectively enhances the survival of plasmablasts generated from human memory B cells. J Clin Invest.

[63] Liu XQ, Stacey KJ, Horne-Debets JM, Cridland JA, Fischer K, Narum D (2012). Malaria infection alters the expression of B-cell activating factor resulting in diminished memory antibody responses and survival. Eur J Immunol.

[64] Capobianco MP, Cassiano GC, Furini AA, Storti-Melo LM, Pavarino EC, Galbiatti AL (2013). No evidence for association of the CD40, CD40 L and BLYS polymorphisms, B-cell co-stimulatory molecules, with Brazilian endemic *Plasmodium vivax* malaria. Trans R Soc Trop Med Hyg.

