# Supplementary material for: Immunogenetic markers associated with a naturally acquired humoral immune response against an N-terminal antigen of Plasmodium vivax merozoite surface protein 1 (PvMSP-1)
Source: Malar J. 2016 Jun 3;15:306. doi: 10.1186/s12936-016-1350-2 (PMC4891883; doi:10.1186/s12936-016-1350-2)
Supplement: Supplementary file 1 — 10.1186/s12936-016-1350-2 Reaction conditions for the amplification and enzyme digestion of polymorphisms in the studied polymorphisms. [file 12936_2016_1350_MOESM1_ESM.docx]

**Additional File 1. Reaction conditions for the amplification and enzyme digestion of polymorphisms in the studied polymorphisms.**

| **Gene** | **SNP** | **Primer5’-3’ (forward)** | **Primer5’-3’ (reverse)** | **Restriction enzyme** | **Fragment length(bp)** |
| --- | --- | --- | --- | --- | --- |
| *CD28* | rs35593994 | TTCTCATTCTGTTGCCCTGGC | CACCATCCCCTTAGGGCACAT | *Hinf*I | G: 468 + 78, A: 546 |
| *CD28* | rs3116496 | GAAACACCTTTGTCCAAGTC | CTCAATGCCTTCTGGGAAATC | *Aci*I | T: 333, C: 193 + 140 |
| *CTLA4* | rs733618 | CTTCATGCCGTTTCCAACTT | CCTTTTCTGACCTGCCTGTT | *Bbv*I | T: 400, C: 210 + 190 |
| *CTLA4* | rs11571316 | CTTCATGCCGTTTCCAACTT | ATCTCCTCCAGGAAGCCTCTT | *Mbo*II | G: 442 + 59 + 26 + 9, A: 275 + 167 + 59 + 26 + 9 |
| *CTLA4* | rs5742909 | GGGATTTAGGAGGACCCTTG | GTGCACACACAGAAGGCACT | *Mse*I | C: 244, T: 179 + 65 |
| *CTLA4* | rs231775 | CTGAACACCGCTCCCATAAA | CACTGCCTTTGACTGCTGAA | *Bbv*I | A: 215, G: 159 + 56 |
| *ICOS* | rs4675378 | TTACCAAGACTTTAGATGCTTTCTT | GAATCTTTCTAGCCAAATCATATTC | *Alu*I | G: 79 + 75 + 58 + 54, A: 154 + 58 + 54 |
| *CD86* | rs1129055 | CTGTTCCAATGGCAACCTCT | GGTTGCCCAGGAACTTACAA | *CviK*I-1 | T: 251 + 195, C: 446 |
| *CD40* | rs1883832 | GAAACTCCTGCGCGGTGAAT | GAAACTCCTGCGCGGTGAAT | *Sty*I | C: 133 + 96 + 74, T: 207 + 96 |
| *BLYS* | rs9514828 | TGGCTCTTGTGTGATCAAGG | GCCTGGTCTCAGCTTTTCTG | *Mbi*I | C: 162 + 48, T: 210 |
