# Supplementary material for: Immunogenetic markers associated with a naturally acquired humoral immune response against an N-terminal antigen of Plasmodium vivax merozoite surface protein 1 (PvMSP-1)
Source: Malar J. 2016 Jun 3;15:306. doi: 10.1186/s12936-016-1350-2 (PMC4891883; doi:10.1186/s12936-016-1350-2)
Supplement: Supplementary file 2 — 10.1186/s12936-016-1350-2 Correlation between ICB2-5-specific IgG, IgM and IgG subclasses in individuals infected with Plasmodium vivax living in an endemic area of the Brazilian Amazon. [file 12936_2016_1350_MOESM2_ESM.docx]

**Additional File 2.** **Correlation between ICB2-5-specific IgG, IgM and IgG subclasses in individuals infected with *Plasmodium vivax* living in an endemic area of the Brazilian Amazon**

|  | **IgG** | **IgG1** | **IgG2** | **IgG3** | **IgG4** |
| --- | --- | --- | --- | --- | --- |
| **IgG** | 1 |  |  |  |  |
| **IgG1** | 0.12 | 1 |  |  |  |
| **IgG2** | 0.16 | 0.48* | 1 |  |  |
| **IgG3** | 0.17 | 0.43* | 0.38* | 1 |  |
| **IgG4** | 0.14 | 0.16 | 0.28* | 0.38 | 1 |
| **IgM** | 0.22* | 0.22 | 0.10 | 0.09 | 0.08 |

*Pearson correlation with *p* < 0.05
