# Supplementary material for: Immunogenetic markers associated with a naturally acquired humoral immune response against an N-terminal antigen of Plasmodium vivax merozoite surface protein 1 (PvMSP-1)
Source: Malar J. 2016 Jun 3;15:306. doi: 10.1186/s12936-016-1350-2 (PMC4891883; doi:10.1186/s12936-016-1350-2)
Supplement: Supplementary file 3 — 10.1186/s12936-016-1350-2 IgM and IgG subclasses levels (RI) for ICB2-5 according to age, length of residence in the studied area and previous episode of malaria. [file 12936_2016_1350_MOESM3_ESM.docx]

|  |  | **IgG1** | |  | **IgG2** | |  | **IgG3** | |  | **IgG4** | |  |  | **IgM** | |
| --- | --- | --- | --- | --- | --- | --- | --- | --- | --- | --- | --- | --- | --- | --- | --- | --- |
| **Epidemiological characteristic** | n | RI^ϯ^ | p |  | RI | p |  | RI | p |  | RI | p |  | n | RI | p |
| Age (years) |  |  | 0.37 |  |  | 0.30 |  |  | 0.46 |  |  | 0.07 |  |  |  | 0.15 |
| 10-20 | 23 | 1.26 (0.76-1.75) |  |  | 0.96 (0.79-1.13) |  |  | 0.79 (0.67-0.90 |  |  | 0.93 (0.73-1.14) |  |  | 42 | 0.95 (0.72-1.19) |  |
| 21-30 | 19 | 1.15 (0.70-1.61) |  |  | 0.90 (0.74-1.05) |  |  | 0.69 (0.60-0.73) |  |  | 0.73 (0.65-0.81) |  |  | 46 | 0.66 (0.45-0.86) |  |
| 31-40 | 21 | 1.00 (0.91-1.09) |  |  | 0.94 (0.73-1.15) |  |  | 0.92 (0.54-1.29) |  |  | 0.71 (0.66-0.76) |  |  | 44 | 0.74 (0.51-0.96) |  |
| >40 | 24 | 0.94 (0.86-1.01) |  |  | 0.85 (0.70-1.01) |  |  | 0.79 (0.69-0.89) |  |  | 0.79 (0.69-0.89) |  |  | 35 | 0.79 (0.56-1.02) |  |
| Length of residence in the studied area |  |  | 0.54 |  |  | 0.10 |  |  | 0.10 |  |  | 0.50 |  |  |  | 0.21 |
| >5 | 38 | 1.10 (0.87-1.32) |  |  | 0.99 (0.85-1.12) |  |  | 0.83 (0.71-0.94) |  |  | 0.80 (0.72-0.88) |  |  | 76 | 0.65 (0.50-0.80) |  |
| 5-10 | 17 | 1.31 (0.63-1.99) |  |  | 0.90 (0.68-1.12) |  |  | 0.87 (0.44-1.29) |  |  | 0.82 (0.56-1.08) |  |  | 29 | 0.68 (0.48-0.88) |  |
| 11-15 | 14 | 0.95 (0.88-1.02) |  |  | 0.82 (0.72-0.91) |  |  | 0.74 (0.61-0.88) |  |  | 0.79 (0.70-0.88) |  |  | 26 | 1.03 (0.66-1.39) |  |
| >15 | 18 | 0.95 (0.84-1.06) |  |  | 0.87 (0.67-1.07) |  |  | 0.68 (0.54-0.82) |  |  | 0.76 (0.63-0.88) |  |  | 36 | 0.92 (0.65-1.19) |  |
| Previous episodes of malaria |  |  | 0.53 |  |  | **0.01** |  |  | 0.69 |  |  | 0.44 |  |  |  | 0.44 |
| <5 | 36 | 1.10 (0.87-1.33) |  |  | 0.97 (0.84-1.10) |  |  | 0.77 (0.66-0.89) |  |  | 0.79 (0.71-0.87) |  |  | 66 | 0.90 (0.69-1.10) |  |
| ≥5 | 49 | 1.08 (0.85-1.31) |  |  | 0.88 (0.76-0.99) |  |  | 0.82 (0.67-0.97) |  |  | 0.80 (0.70-0.90) |  |  | 87 | 0.73 (0.60-0.87) |  |

**Additional File 3. IgM and IgG subclasses** **levels (RI) for ICB2-5 according to age, length of residence in the studied area and previous episode of malaria.**

ϯ RI expressed as median (95% CI).
