# Supplementary material for: Immunogenetic markers associated with a naturally acquired humoral immune response against an N-terminal antigen of Plasmodium vivax merozoite surface protein 1 (PvMSP-1)
Source: Malar J. 2016 Jun 3;15:306. doi: 10.1186/s12936-016-1350-2 (PMC4891883; doi:10.1186/s12936-016-1350-2)
Supplement: Supplementary file 4 — 10.1186/s12936-016-1350-2 Association tests between genetic polymorphisms and Plasmodium vivax ICB2-5-specific antibody levels. [file 12936_2016_1350_MOESM4_ESM.docx]

| **Gene** | **SNP** | **Allele**  **(MAF^a^)** | **IgG**  **(p^b^)** | **IgG1**  **(p)** | **IgG2**  **(p)** | **IgG3**  **(p)** | **IgG4**  **(p)** | **IgM**  **(p)** |
| --- | --- | --- | --- | --- | --- | --- | --- | --- |
| *CD28* | rs35593994 | A (0.292) | 0.44 | 0.42 | 0.35 | 0.20 | 0.19 | **0.033^c^** |
| *CD28* | rs3116496 | C (0.185) | 0.35 | **0.00004^d^** | 0.69 | 0.36 | 0.41 | 0.30 |
| *CTLA4* | rs733618 | C (0.076) | 0.23 | 0.44 | **0.042^e^** | 0.28 | 0.18 | 0.26 |
| *CTLA4* | rs11571316 | A (0.339) | 0.46 | 0.56 | 0.20 | 0.23 | 0.10 | 0.77 |
| *CTLA4* | rs5742909 | T (0.076) | 0.15 | 0.18 | 0.83 | 0.73 | 0.93 | 0.81 |
| *CTLA4* | rs231775 | G (0.343) | 0.31 | 0.12 | 0.19 | 0.51 | 0.84 | 0.66 |
| *ICOS* | rs4675378 | C (0.359) | 0.34 | 0.50 | 0.36 | 0.07 | 0.92 | 0.31 |
| *CD86* | rs1129055 | A (0.213) | 0.14 | 0.78 | 0.80 | 0.73 | 0.16 | 0.38 |
| *CD40* | rs1883832 | T (0.138) | 0.26 | 0.50 | 0.23 | 0.44 | 0.46 | 0.21 |
| *BLYS* | rs9514828 | T (0.247) | 0.18 | 0.16 | 0.64 | 0.11 | 0.15 | 0.14 |

**Additional File 4 Association tests between genetic polymorphisms and *Plasmodium vivax* ICB2-5-specific antibody levels**

^a^Minor allele frequency

^b^p-values based on fitting linear regression models adjusting for age and gender

^c^Dominant model

^d^Recessive model

^e^Overdominant model
